# Idiopathic Sclerosing Encapsulating Peritonitis Associated With Persistent Descending Mesocolon: A Surgical Puzzle

**DOI:** 10.7759/cureus.45679

**Published:** 2023-09-21

**Authors:** Anna Aourelia Maria Skarmoutsou, Antonio Pujante Antonatou, Themistoklis Zekeridis, Aliki Fiska

**Affiliations:** 1 1st General Surgery Department, University Hospital of Alexandroupolis, Alexandroupoli, GRC; 2 General Surgery, George Papanikolaou General Hospital, Thessaloniki, GRC; 3 General and Colorectal Surgery, General Hospital of Kavala, Kavala, GRC; 4 Laboratory of Anatomy, Faculty of Medicine, Democritus University of Thrace, Alexandroupoli, GRC

**Keywords:** encapsulating peritoneal sclerosis, abdominal cocoon syndrome (acs), emergency surgery, intestine necrosis, sclerosing encapsulating peritonitis (sep), persistent descending mesocolon

## Abstract

During our practice as clinical surgeons, we have encountered situations in which exploratory abdominal laparotomies have yielded unexpected outcomes, despite conducting thorough and rigorous preoperative studies. A rare condition called sclerosing encapsulating peritonitis (SEP), in which a fibrocollagenous membrane encircles the intestine and other abdominal organs, surprised us in a case of an acute abdomen. Persistent descending mesocolon is another unusual condition in which the descending colon is transferred downward and to the right abdominal region because its mesocolon is unable to merge with the posterior abdominal wall. Those two different conditions are extremely rare and were never been described in a single case. We present a case of an 80-year-old male who presented in the emergency department with an acute abdomen and puzzled us.

## Introduction

Despite meticulous preoperative investigation, exploratory abdominal laparotomy may present with unexpected findings, as several reported surgical cases indicate. Sclerosing encapsulating peritonitis (SEP) is one of the rare conditions in which a fibrocollagenous membrane forms in the abdomen, wrapping around parts of the bowel and other organs. This anatomical disorganization may eventually cause intestinal obstruction and present with signs and symptoms of acute abdomen [1].

Persistent descending mesocolon is another extraordinary disorder, the descending colon fails to fuse with the posterior abdominal wall and is transposed down and to the right abdominal area. It remains suspended by the mesocolon, but lies beneath the small intestine, along with parts of the drifted-away distal transverse colon. This is clearly due to the colon’s failure to normally 270°^ ^counter-clockwise rotate around the superior mesenteric pedicle, during embryologic development. After assuming the normal position, the colon surrounds the small intestine and inhibits its escape to other anatomical positions.

We report a case, which greatly puzzled us during exploratory laparotomy for acute abdomen, of the coexistence of these two rare pathologies - sclerosis encapsulating peritonitis (SEP) and persistent descending mesocolon. Both are uncommon developmental abnormalities of the lower gastrointestinal tract and, to our knowledge, they have not previously been reported to occur simultaneously.

## Case presentation

An 80-year-old male was brought to the emergency department with epigastric pain of acute onset, gradually worsening for two days, accompanied by nausea, vomiting, and subfebrile temperature (38°C). The patient’s medical record included hypertension, rheumatoid arthritis, and hyperlipidemia, but he had no history of abdominal pathology or prior surgery.

Physical examination revealed upper abdominal distension, tenderness to palpation, and tympanic sound on percussion, but no signs of general peritonitis. Laboratory analysis demonstrated a white blood count (WBC) of 21.200 K/μL, c-reactive protein (CRP) value of 28.72 mg/dL, and mild hypoalbuminemia (2.7 mg/dL). Chest and abdominal x-ray and abdominal ultrasound showed no specific findings.

**Figure 1:**
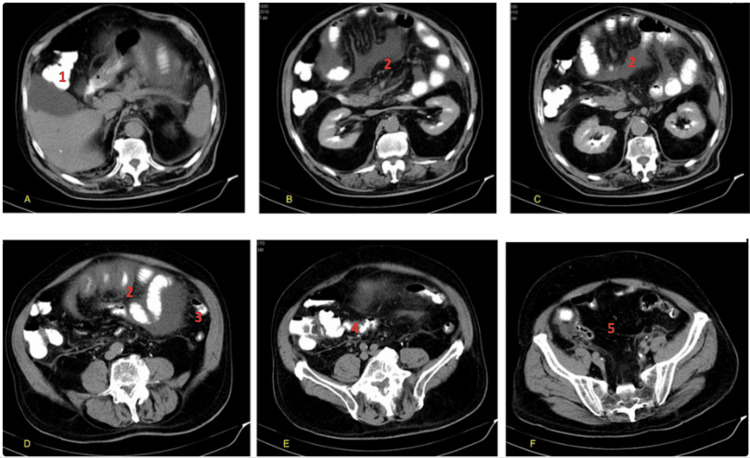
CT scan showcasing SEP and persistent descending mesocolon (A-F). SEP: sclerosing encapsulating peritonitis The images show (1) right colic flexure, (2) thickened and dilated small bowel loops located in the epigastrium, (3) splenic flexure, and (4) transverse colon located below the small bowel and (5) pelvis. No small bowel loops were found in the iliac fossa (only cecum is visible).

The CT scan showed a large, circumscribed mass, occupying the epigastrium and most of the left hypochondrium and lumbar region. It consisted of a membrane-encased cluster of thickened and dilated small bowel loops, with interloop ascites. The mass displaced the transverse colon to the right and lower abdominal area, giving the unusual impression of small intestine lying over the third and fourth portion of the colon (Figures 1A-1F).

**Figure 2:**
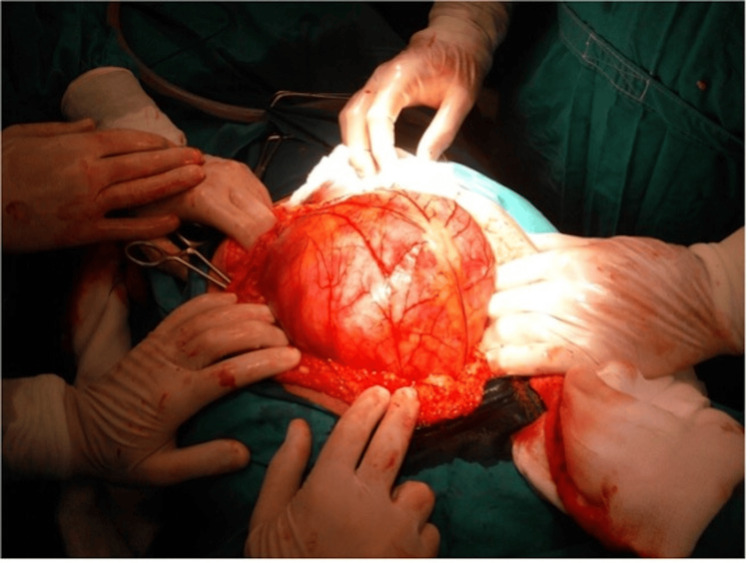
Sac containing intestinal loops.

The treatment of choice was emergency exploratory laparotomy, due to the patient’s gradually deteriorating status and abnormal CT findings. As soon as we entered the peritoneal cavity, we faced a large translucent, highly vascularized sac approximately 20 cm in diameter, projecting through the incision and containing several intestinal loops (Figure 2).

The sac was located between the stomach (superiorly), the spleen (to the left), and the transverse and descending colon and mesocolon (to the right and below). The cecum with the ileocolic junction and the right colon (including the proximal portion of the transverse colon) were greatly distended but remained in their anatomic location. The rest of the transverse colon and the distal parts of the large intestine appeared collapsed, yet the transition point was free of obstruction signs (either inflammatory, neoplastic, or adhesive).

**Figure 3:**
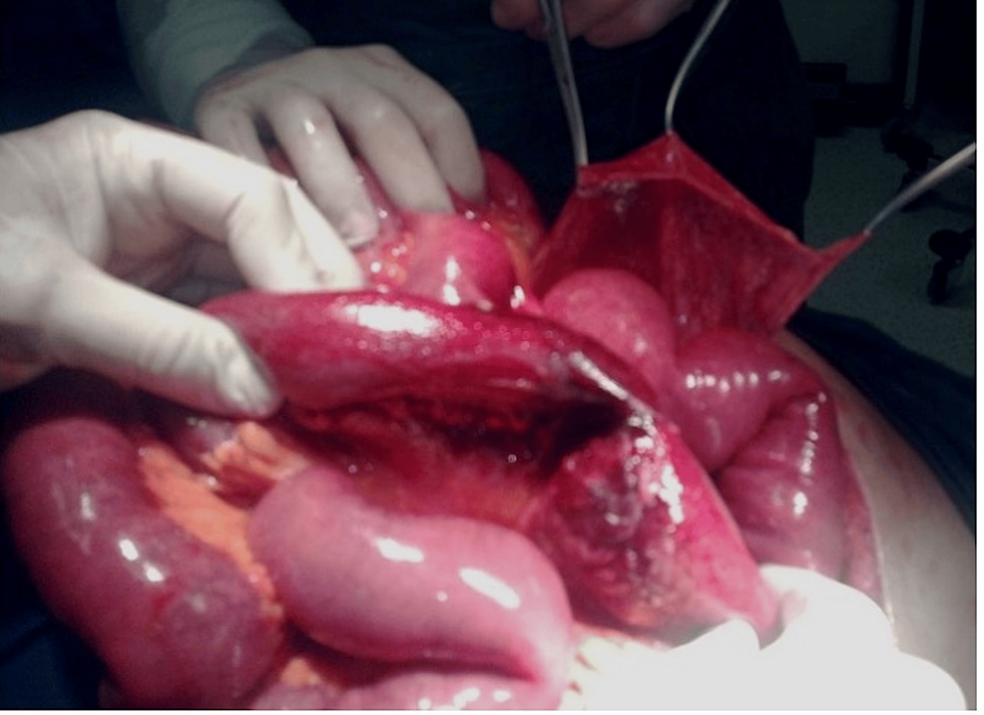
Small intestine found as content of the sac.

To unravel the enigma, we followed the course of the terminal ileum from the ileocolic junction up. We could only measure approximately 10 cm of terminal ileum, before losing it through an opening of the transverse mesocolon. This opening seemed to connect the greater sac of the peritoneum with the interior of the abnormal sac. The sac’s content comprised the rest of the ileum and all the jejunum, up to the ligament of Treitz. All the loops were markedly distended. On thorough examination, we noticed a necrotic section, about 20 cm proximal to the entrance point of the intestine through the mesocolic opening, which we resected and closed the mesocolic opening (Figure 3). Before surgical closure, we tried to remove as much of the remaining sac as possible.

Cytology of a sample of ascetic fluid and pathological evaluation of the resected ileum and a sample of the sac did not show evidence of neoplastic disease. The patient remained in the ICU for 41 days, during which he presented multiple atrial fibrillation episodes and was mostly nourished via a nasogastric tube. On the 36th day, the patient went back to automatic breathing. On the 41st day, he was transferred to our surgical department and 15 days later he was dismissed. The one-year follow-up was complication-free. He died after eight years due to an ischemic stroke attack.

## Discussion

Sclerosing encapsulating peritonitis (SEP) has been given various names since it was introduced by Owtschinnikow in 1907 as “peritonitis chronica fibrosa incapsulata.” In 1978, Foo et al. introduced the term "abdominal cocoon syndrome" to describe its idiopathic form. Various names have been used over the years to describe it, such as icing sugar bowel, fibroplastic peritonitis, and peritoneal encapsulation. Apparently, the most appropriate term is encapsulating peritoneal sclerosis, because it excludes the word peritonitis, as it implies inflammation [2-4].

SEP presents with two entities as follows: secondary, which is the most common type, correlated with other chronic fibrosing inflammatory conditions, such as tuberculosis, sarcoidosis, peritoneal dialysis, pelvic inflammatory disease, autoimmune disease, and hepatitis C virus infection [1-4]. Idiopathic or primary, with unknown pathogenesis and no associated underlying condition, which is extremely rare and is the case of our patient.

The clinical diagnosis is difficult, because of the non-specific manifestations. Most of the patients present with symptoms of subacute intestinal obstruction, such as abdominal pain or distention, and in fewer cases with acute abdomen [1]. Preoperative identification is now possible because of the evolution of radiology, but the definite diagnosis is still sometimes set in the operation theatre [4].

The typical radiological sign of SEP in a computer tomography (CT) scan is the image of a bunch of intestinal loops enclosed in a thickened sheath with continuous enhancement, whereas the normal peritoneum is barely visible and appears with discontinuous enhancement. Signs of obstruction may be present. The CT scan is additionally valuable for investigating the cause of a suspected secondary SEP. Abdominal ultrasonography and MRI can provide important clues as well [3].

Differential diagnosis includes internal hernias into the lesser sac, that can occur through the foramen of Winslow, through a defect in the lesser or greater omentum, or through a defect in the transverse mesocolon. The latter is usually secondary to abdominal trauma or previous surgery [5]. The sac of these hernias is formed by the margins of the lesser sac. In our case, there was no doubt that this was not an internal transmesenteric hernia since its walls were well-defined and clearly distinct from the adjacent transverse mesocolon and the lesser omentum.

Surgical treatment remains the gold standard for symptomatic patients. The therapeutic approach is surgical excision of the membrane and adhesiolysis [2]. If the bowel appears affected and non-viable, resection with primary anastomosis or stoma creation is indicated [1,4]. Conservative management has been found to be efficacious for cases with mild symptomatology [3]. This includes the use of anti-inflammatory agents including colchicine and immunosuppressants [3].

Several reports have suggested the correlation of idiopathic SEP with congenital anomalies, such as the absence of the greater omentum and gastrocolic ligament. Less commonly associated with it are hernia, accessory sac, and cryptorchidism [1]. Notably, all these conditions fall in the category of developmental disorders that refer to the embryological arrangement of the peritoneum in the abdominal cavity.

In our patient, as is usually the case, the diagnosis of SEP was made when he presented with paroxysmal abdominal pain, due to obstruction. What was remarkable is that it brought to light an even rarer simultaneous congenital variant of the gastrointestinal tract, that has never been reported to accompany SEP.

**Figure 4:**
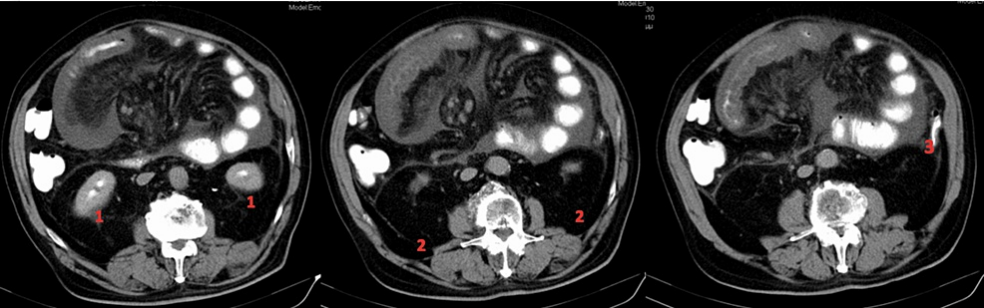
CT scan showing the lower position of the splenic flexure. The images show (1) the lower poles of the right and left kidneys, (2) perirenal adipose tissue found under the kidneys, and (3) left colic flexure (splenic flexure).

The sac containing the small bowel was found adjacent to the left abdominal wall, displacing the transverse and descending colon lower and to the right. This location of the sac could only be possible due to an aberrant positioning of the distal half of the transverse and the descending colon, in combination with the lowering of the left colic flexure (it was found right below the lower pole of the left kidney) (Figure 4).

The condition indicated persistent descending mesocolon, which represents the failure of the mesentery of the left colon to fuse with the posterior abdominal wall during fetal development, in the fifth month of gestation. As a result, the descending colon remains suspended by its mesocolon, and its location varies greatly. It commonly repositions medially, carrying away the left colic flexure and the distal transverse colon. This rearrangement of the colon leaves an area of the left abdomen free of viscera (the paracolic fossa) into which the small bowel may migrate. The ileocecal junction rests in the right iliac fossa, and the small intestine finds its way to it, passing right in front of the descending colon or, as in our case, through an opening in the mesocolon [6,7].

The condition is usually asymptomatic, because of the short height of the mesocolon and is accidentally diagnosed during routine examinations or an investigation of another pathology. Rarely, intestinal obstruction may be caused by colon twisting or internal hernia, when a long mesocolon is present [6].

**Figure 5:**
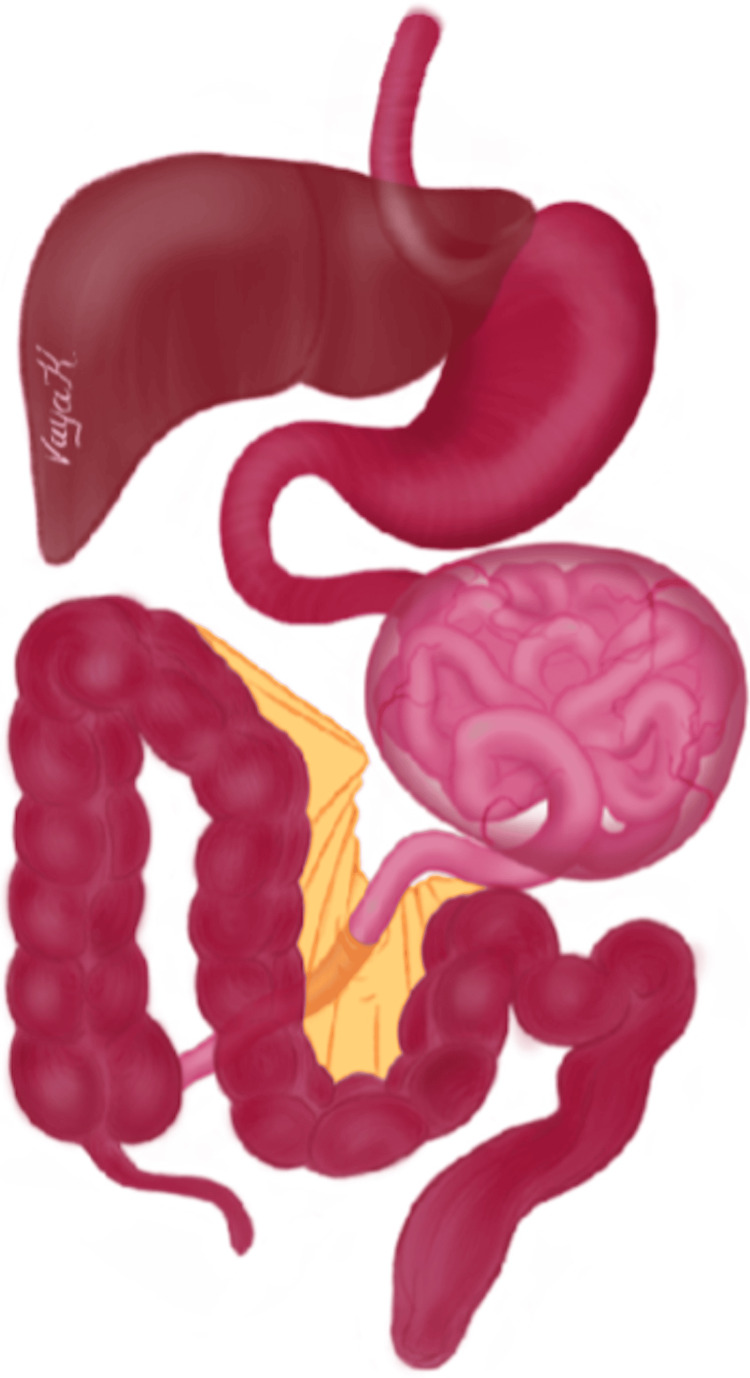
Schematic representation of the combination of SEP and persistent descending mesocolon. SEP: sclerosing encapsulating peritonitis The image has been created by Dr. Vaya Karapepera. Permission has been obtained for its reproduction.

The obvious question is if the lower transposed splenic flexure of our patient fits the diagnosis of persistent descending mesocolon or if it is just a malrotation of the gastrointestinal tract that ended up with the small bowel pushing the left colon in a lower position. The latter possibility means that the intestinal migration and its peritoneal encapsulation occurred before the fifth gestational month and that he had lived with it, free of symptoms, for 80 years. Nevertheless, it is still a rare developmental deformity of the gastrointestinal tract, exceptionally associated with idiopathic SEP (Figure 5).

In any case, as it is important for clinical doctors, especially for surgeons to raise awareness of the existence of SEP, it is equally imperative to know that it can be associated with other, mostly innate, anatomical deviations. The preoperative suspicion can help determine the diagnosis, define the correct management, and most importantly protect the surgeon from clearly unpleasant surprises on the operating table.

## Conclusions

Sclerosing encapsulating peritonitis (SEP) and persistent descending mesocolon, two uncommon and separate pathological diseases, coexist in the case presented in this report, creating a unique and difficult therapeutic setting. The simultaneous presence of both of these uncommon lower gastrointestinal tract conditions is unheard of in the medical literature. The presence of SEP and its potential relationships with other congenital anatomical anomalies must be understood in therapeutic practice.
